# Interactive computations: toward risk management in interactive intelligent systems

**DOI:** 10.1007/s11047-015-9486-5

**Published:** 2015-02-11

**Authors:** Andrzej Skowron, Andrzej Jankowski

**Affiliations:** 1 Institute of Mathematics, University of Warsaw, Banacha 2, 02-097 Warsaw, Poland; 2 Knowledge Technology Foundation, Nowogrodzka 31, 00-511 Warsaw, Poland; 3 Systems Research Institute PAS, Newelska 6, 01-447 Warsaw, Poland

**Keywords:** Rough sets, Granular computing, Interactive computations, Interactive intelligent systems, Risk management, Wisdom Technology, 68T05, 68T27, 68T37

## Abstract

Understanding the nature of interactions is regarded as one of the biggest challenges in projects related to complex adaptive systems. We discuss foundations for interactive computations in interactive intelligent systems (IIS), developed in the Wistech program and used for modeling complex systems. We emphasize the key role of risk management in problem solving by IIS. The considerations are based on experience gained in real-life projects concerning, e.g., medical diagnosis and therapy support, control of an unmanned helicopter, fraud detection algorithmic trading or fire commander decision support.

## Introduction

Information granules (infogranules, for short) are widely discussed in the literature (see, e.g., Pedrycz et al. 2008). In particular, let us mention here the rough granular computing approach based on the rough set approach and its combination with other approaches to soft computing. However, the issues related to interactions of infogranules with the physical world and to perception of interactions in the physical world represented by infogranules are not well elaborated yet. On the other hand the understanding of interactions is the critical issue of complex systems (Goldin et al. 2006) in which computations are progressing by interactions among information granules and physical objects.

We extend the existing approach to granular computing by introducing *complex granules* (c-*granules*, for short) (Jankowski 2015) making it possible to model interactive computations performed by agents in interactive intelligent systems (IIS) used for modeling of complex systems.

Any agent operates in a local world of c-granules. The agent control is aiming to control computations performed on c-granules from this local world for achieving the target goals.

Computations in IIS are based on c-granules. The risk management in IIS is of the great importance for the success of behaviors of individuals, groups and societies of agents. The risk management tasks are considered as control tasks aiming at achieving the satisfactory performance of (societies of) agents. The novelty of the proposed approach is the use of complex vague concepts as the guards of control actions. These vague concepts are represented, e.g., using domain ontologies. The rough set approach in combination with other soft computing approaches is used for approximation of the vague concepts relative to attributes (features) available to the risk management systems.

This paper is organized as follows. In Sect. 2 an introduction to Interactive Rough Granular Computing (IRGC) is presented. Issues related to reasoning based on adaptive judgment are included in Sect. 3. In Sect. 4 the relatonships of complex granules with the satisfiability relations are outlined. The approach to risk management based on IRGC is discussed in Sect. 6.

This paper covers some issues presented in the plenary talk at the 5th International Conference on Pattern Recognition and Machine Intelligence (PReMi 2013), December 10–14, 3013, Kolkata, India and is a summarization and an extension of Skowron et al. (2012), Jankowski et al. (2013, 2014a, 2014b). Interactive computations on c-granules may be used for modeling computations in Natural Computing Kari and Rozenberg (2008), Rozenberg et al. (2012), Ehrenfeucht et al. (2012). This issue is discussed in the paper in more detail.

## Interactive rough granular computing (IRGC)

The essence of the proposed approach is the use of IIS implemented using IRGC (Jankowski and Skowron 2009; Skowron and Wasilewski 2011, 2012; Skowron et al. (2012; Jankowski 2015; Skowron et al. 2012). The approach is based on foundations for modeling of IRGC relevant for IIS in which computations are progressing through interactions (Goldin et al. 2006). In IRGC interactive computations are performed on objects called *complex granules* (*c-granules*, for short) linking information granules (Pedrycz et al. 2008) (or infogranules, for short) with physical objects called hunks (Heller 1990; Jankowski 2015).

Infogranules are widely discussed in the literature. They can be treated as specifications of compound objects (such as complex hierarchically defined attributes) together with scenarios of their implementations. Such granules are obtained as the result of information granulation (Zadeh 2001):

Information granulation can be viewed as a human way of achieving data compression and it plays a key role in implementation of the strategy of divide-and-conquer in human problem-solving.

Infogranules belong to the concepts playing the main role in developing foundations for AI, data mining and text mining (Pedrycz et al. 2008). They grew up as some generalizations from fuzzy sets (Zadeh 1979, 1999, 2001), rough set theory and interval analysis (Pedrycz et al. 2008). The rough set approach is crucial because of necessity to deal with approximations of infogranules by the others, e.g., in inducing classifiers for complex vague concepts. The IRGC is based on the rough set approach in combination with other approaches to soft computing (such as fuzzy sets). However, the issues related to interactions of infogranules with the physical world and their relation to perception of interactions in the physical world are not well elaborated yet (Goldin et al. 2006; Vapnik 1998). On the other hand the understanding of interactions is the critical issue of complex systems (Omicini 2006):

[...] interaction is a critical issue in the understanding of complex systems of any sorts: as such, it has emerged in several well-established scientific areas other than computer science, like biology, physics, social and organizational sciences.

**Fig. 1:**
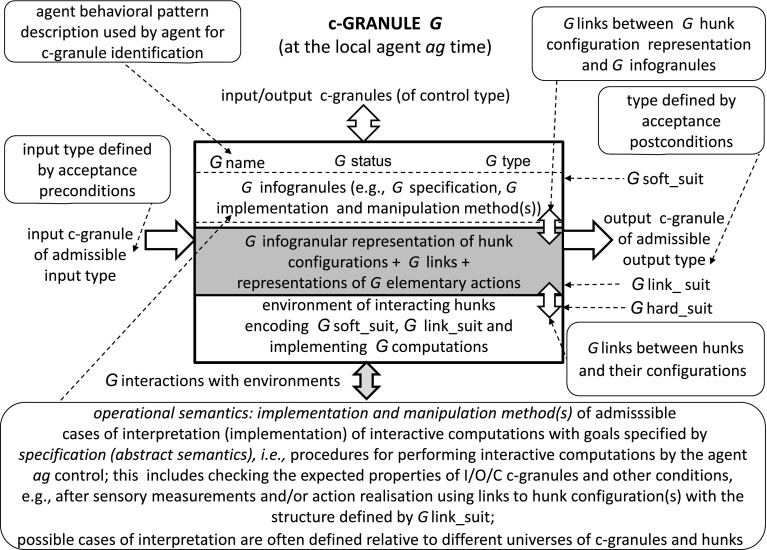
General structure of c-granule

We propose to model complex systems by IIS created by societies of agents. Computations in the discussed IIS are based on c-granules (Jankowski 2015) (see Fig. 1). Any c-granule consists of three components, namely soft_suit, link_suit and hard_suit. These components are making it possible to deal with such abstract objects from soft_suit as infogranules as well as with physical objects from hard_suit. The link_suit of a given c-granule is used as a kind of c-granule interface for handling interaction between soft_suit and and hard_suit.

**Fig. 2:**
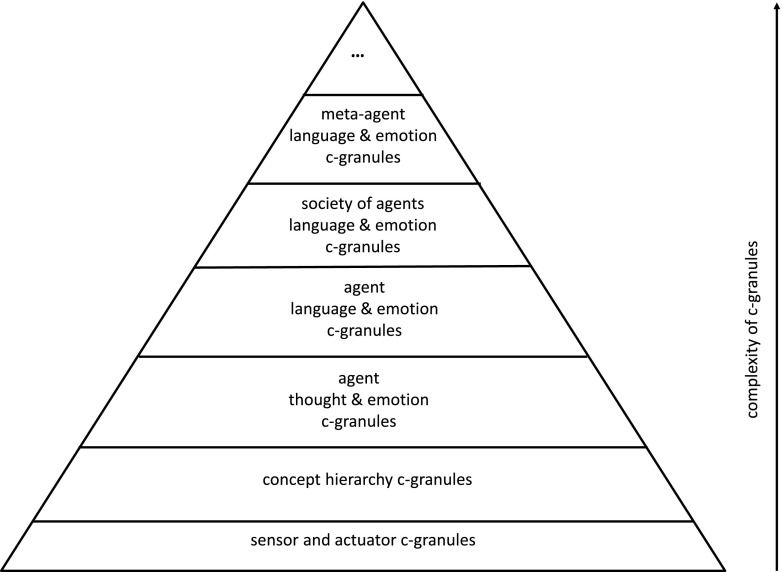
Hierarchy of c-granules

Calculi of c-granules are defined by elementary c-granules (defined, e.g., by indiscernibility of similarity classes) and c-granules making it to possible to generate new c-granules from already defined ones (see Fig. 1) where the presented c-granule produces new output c-granules from the given input c-granules. The hierarchy of c-granules is illustrated in Fig. 2. Moreover, c-granules create the basis for the agent (communication) language construction and the language evolution.

Any agent operates in a local world of c-granules. The agent control is aiming to control computations performed on c-granules from this local world for achieving the target goals. Actions (sensors or plans) from link_suits of c-granules are used by the agent control in exploration and/or exploitation of the environment on the way to achieve the agent targets. C-granules are also used for representation of perception by agents of interactions in the physical world. Due to the bounds of the agent perception abilities usually only a partial information about the interactions from physical world may be available for agents. Hence, in particular the results of performed actions by agents can not be predicted with certainty. For more details on IRGC based on c-granules the reader is referred to Jankowski (2015).

One of the key issues of the approach to c-granules presented in Jankowski (2015) is some kind of integration of investigation of physical and mental phenomena. The integration follows from suggestions presented by many scientists. For illustration let us consider following two quotations strongly related to the research on IRGC based on c-granules:

As far as the laws of mathematics refer to reality, they are not certain; and as far as they are certain, they do not refer to reality.

– Albert Einstein (Einstein 1921)

Constructing the physical part of the theory and unifying it with the mathematical part should be considered as one of the main goals of statistical learning theory.

– Vladimir Vapnik (Vapnik 1998, p. 721)

A special role in IRGC play information (decision) systems from the rough set approach (Pawlak 1982, 1991; Pawlak and Skowron 2007; Stepaniuk 2008). They are used to record processes of interacting configurations of hunks. In order to represent interactive computations (used, e.g., in searching for new features) information systems of a new type, namely interactive information systems, are needed (Skowron and Wasilewski 2011, 2012; Jankowski 2015).

## Adaptive Judgment

The reasoning making it possible to derive relevant information granules for solutions of the target tasks is called *adaptive judgment*. *Intuitive judgment* and *rational judgment* are distinguished as different kinds of Kahneman (2002). Among the tasks for adaptive judgment are the following ones supporting reasoning toward: searching for relevant approximation spaces, discovery of new features, selection of relevant features (attributes), rule induction, discovery of inclusion measures, strategies for conflict resolution, adaptation of measures based on the minimum description length principle, reasoning about changes, selection of parameters of (action and sensory) attributes, adaptation of quality measures over computations relative to agents, adaptation of object structures, discovery of relevant context, strategies for knowledge representation and interaction with knowledge bases, ontology acquisition and approximation, learning in dialogue of inclusion measures between information granules from different languages (e.g., the formal language of the system and the user natural language), strategies for adaptation of existing models, strategies for development and evolution of communication language among agents in distributed environments, strategies for risk management in distributed computational systems.

**Fig. 3:**
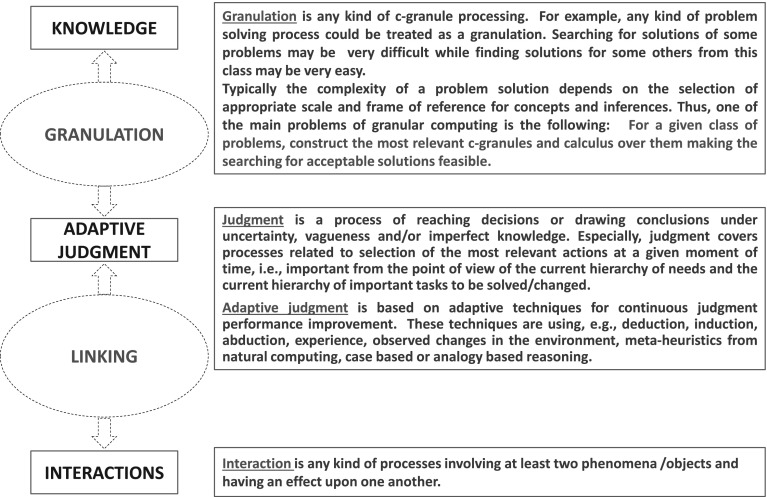
Interactions, adaptive judgment and granulation

Adaptive judgment in IIS is a mixture of reasoning based on deduction, abduction, induction, case based or analogy based reasoning, experience, perceived changes in the environment. Meta-heuristics from natural computing are used to support judgment (see Fig. 3). Let us also note the following remark (Thiele 2010):

Practical judgment is not algebraic calculation. Prior to any deductive or inductive reckoning, the judge is involved in selecting objects and relationships for attention and assessing their interactions. Identifying things of importance from a potentially endless pool of candidates, assessing their relative significance, and evaluating their relationships is well beyond the jurisdiction of reason.

We would like to stress that still much more work should be done to develop approximate reasoning methods about complex vague concepts for making progress in development of IIS, in particular for the risk management in IIS. This idea was very well expressed by Leslie Valiant:^1^

A fundamental question for artificial intelligence is to characterize the computational building blocks that are necessary for cognition. A specific challenge is to build on the success of machine learning so as to cover broader issues in intelligence. [...] This requires, in particular a reconciliation between two contradictory characteristics – the apparent logical nature of reasoning and the statistical nature of learning.

It is worthwhile to mention two more views. The first one by Lotfi A. Zadeh, the founder of fuzzy sets and the computing with words paradigm (see Zadeh (1999) and also http://www.cs.berkeley.edu/~zadeh/presentations.html):

Manipulation of perceptions plays a key role in human recognition, decision and execution processes. As a methodology, computing with words provides a foundation for a computational theory of perceptions - a theory which may have an important bearing on how humans make - and machines might make - perception-based rational decisions in an environment of imprecision, uncertainty and partial truth. [...] computing with words, or CW for short, is a methodology in which the objects of computation are words and propositions drawn from a natural language.

and the view by Judea Pearl included as the motto of this paper.

**Fig. 4:**
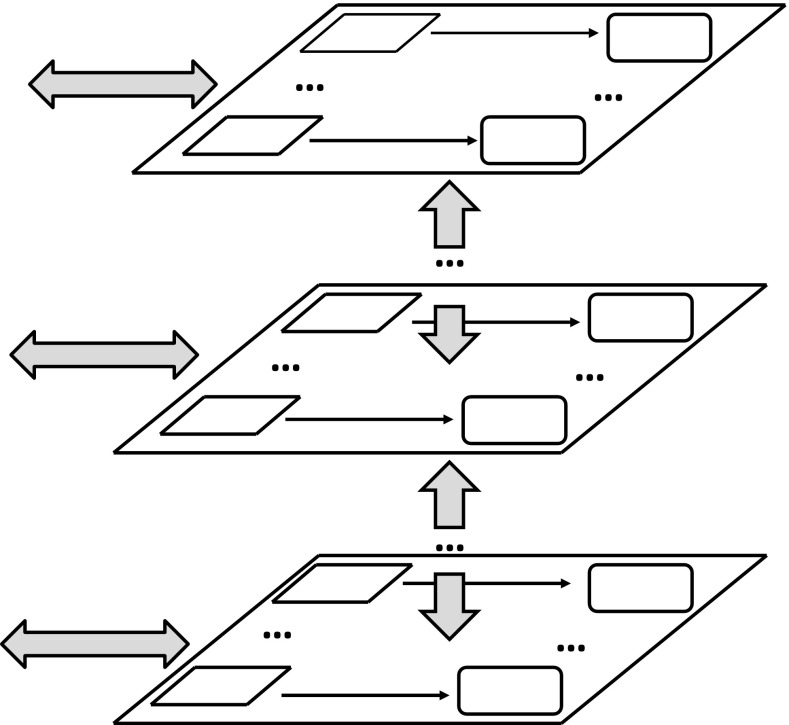
Interactive hierarchical structures (*gray arrows* show interactions between hierarchical levels and the environment, *arrows* at hierarchical levels point from information (decision) systems representing partial specifications of satisfiability relations to induced from them theories consisting of rule sets)

The question arises about the logic relevant for the above discussed tasks. First let us observe that the satisfiability relations in the IRGC framework can be treated as tools for constructing new information granules. If fact, for a given satisfiability relation, the semantics of formulas relative to this relation is defined. In this way the candidates for new relevant information granules are obtained. We would like to emphasize a very important feature. The relevant satisfiability relation for the considered problems is not given but it should be induced (discovered) on the basis of a partial information encoded in information (decision) systems. For real-life problems, it is often necessary to discover a hierarchy of satisfiability relations before we obtain the relevant target level. Information granules constructed at different levels of this hierarchy finally lead to relevant ones for approximation of complex vague concepts related to complex information granules expressed in natural language (see Fig. 4).

## Complex Granules and Satisfiability

**Fig. 5:**
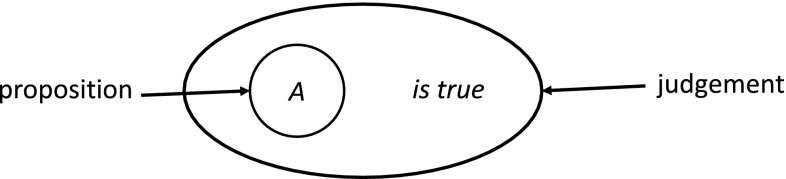
“When we hold a proposition to be true, then we make a judgment.” (Martin-Löf 1984)

In this section, we discuss some examples of c-granules constructed over a family of satisfiability relations being at the disposal of a given agent. This discussion has some roots in intuitionism (see, e.g., Martin-Löf 1984). Let us consider a remark made by Per Martin-Löf in Martin-Löf (1984) about judgment presented in Fig. 5.

In the approach based on c-granules, the judgment for checking values of descriptors (or more compound formulas) pointed by links from simple c-granules is based on interactions of some physical parts considered over time and/or space (called hunks) and pointed by links of c-granules. The judgment for the more compound c-granules is defined by a relevant family of procedures also realized by means of interactions of physical parts.

Let us explain in more detail the above claims.

Let assume that a given agent $ag$ has at the disposal a family of satisfiability relations

1 $$\begin{aligned} \{\models _i \}_{i\in I}, \end{aligned}$$

where $\models _i \subseteq Tok(i)\times Typ(i)$, $Tok(i)$ is a set of tokens and $Type(i)$ is a set of types, respectively (using the terminology from Barwise and Seligman 1997). The indices of satisfiability relations are vectors of parameters related, e.g., to time, space, spatio-temporal features of physical parts represented by hunks or actions (plans) to be realized in the physical world.

In the discussed example of elementary c-granules, $Tok(i)$ is a set of hunks and $Type(i)$ is a set of descriptors (elementary infogranules), respectively, pointed by link represented by $\models _{i}$. The procedure for computing the value of $h \models _{i} \alpha$, where $h$ is a hunk and $\alpha$ is an infogranule (e.g., descriptor or formula constructed over descriptors) is based on interaction of $\alpha$ with the physical world represented by hunk $h$.

The agent control can aggregate some simple c-granules into more compound c-granules, e.g., by selecting some constraints on subsets of $I$ making it possible to select relevant sets of simple c-granules and consider them as a new more compound c-granule. In constraints also values in descriptors pointed by links in elementary c-granules can be taken into account and sets of such more compound c-granules can be aggregated into new c-granules. Values of new descriptors pointed by links of these more compound granules are computed by new procedures. The computation process again is realized by interaction of the physical parts represented by hunks pointed by links of elementary c-granules included in the considered more compound c-granule as well as by using the procedure for computing of values of more compound descriptors from values of descriptors included in elementary c-granules of the considered more compound c-granule. Note that this procedure is also realized in the physical world thanks to relevant interactions.

In hierarchical modeling aiming at inducing of relevant c-granules (e.g., for approximation of complex vague concepts), one can consider so far constructed c-granules as tokens. For example, they can be used to define structured objects representing corresponding hunks and link them using new satisfiability relations (from a given family) to relevant higher order descriptors together with the appropriate procedures (realized by interactions of hunks) for computing values of these descriptors. This approach generalizes hierarchical modeling developed for infogranules (see, e.g., Nguyen et al. 2004; Bazan 2008) to hierarchical modeling of c-granules which is important for many real-life projects.

**Fig. 6:**
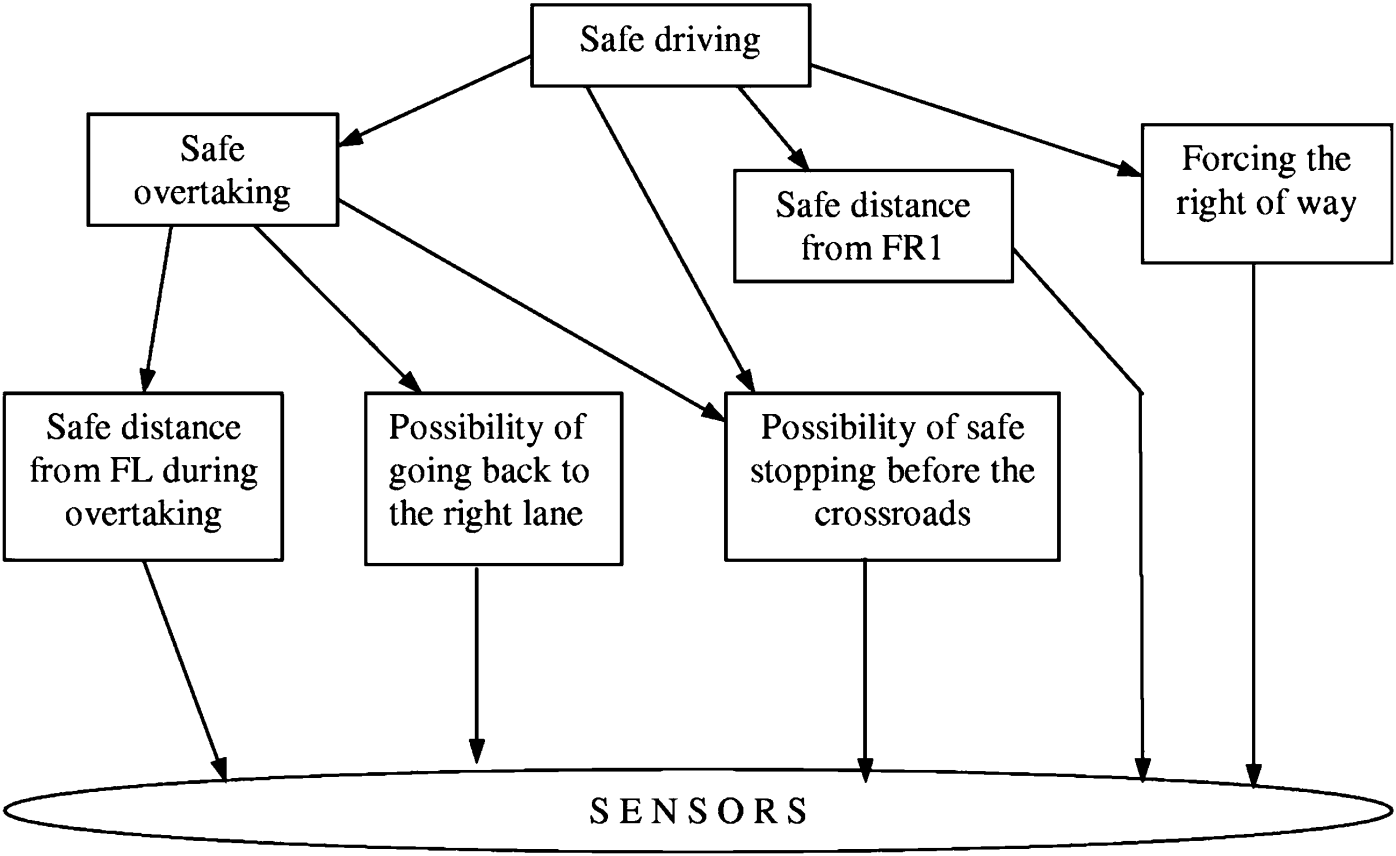
Fragment of ontology used for approximation of the vague concept *safe driving* for decision support in traffic control

**Fig. 7:**
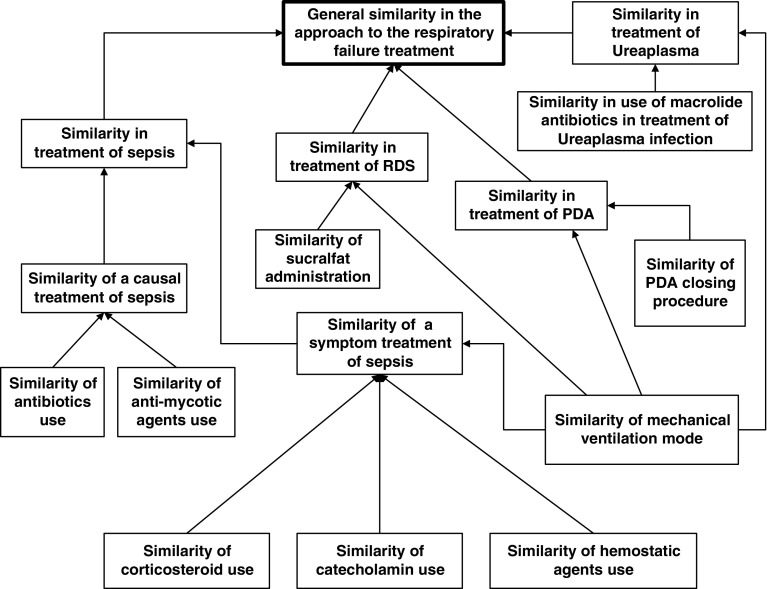
Fragment of ontology used for approximation of the vague concept *similarity* in decision support for respiratory failure

We have assumed before that the agent $ag$ is equipped with a family of satisfiability relations. However, in real-life projects the situation is more complicated. The agent $ag$ should have strategies for discovery of new relevant satisfiability relations on the way of searching for solutions of target goals (problems). This is related to a question about the adaptive judgment relevant for agents performing computations based on configurations of c-granules. In the framework of granular computing based on c-granules, satisfiability relations are tools for constructing new c-granules. In fact, for a given satisfiability relation, the semantics of descriptors (and more compound formulas) relative to this relation can be defined. In this way candidates for new relevant c-granules are obtained. Hence, there arises a very important issue. The relevant satisfiability relations for the agent $ag$ searching for solutions of problems are not given but they should be induced (discovered) on the basis of a partial information encoded in information (decision) systems including results of measurements of interaction of parts of physical world pointed by links of elementary c-granules as well as on the basis of c-granules representing domain knowledge. This problem is strongly related to reasoning from sensory measurement to perception Zadeh (1999), Zadeh (2001). For real-life problems, it is often necessary to discover a hierarchy of satisfiability relations before the relevant target level will be obtained (see Fig. 4) (Jankowski et al. 2013). C-granules constructed at different levels of this hierarchy finally lead to relevant c-granules (e.g., for approximation of complex vague concepts) expressed very often in natural language. This is illustrated in Figs. 6 and 7, where the complex vague concepts *safe driving* and *similarity in respiratory failure* are represented, respectively, together with concepts and relations from fragments of domain ontologies used as hints in searching for relevant attributes (features) on different levels of the hierarchy. For details, the reader is referred to Nguyen et al. (2004), Bazan (2008).

## Agent and Interactive Computations on Complex Granules

**Fig. 8:**
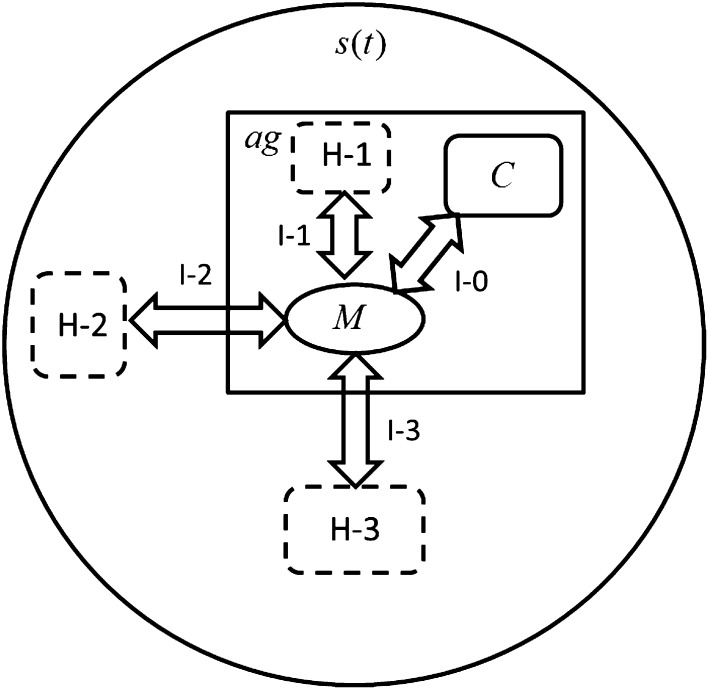
Basic agent components for interactions

In Fig. 8 are illustrated the basic agent components for interactions such as control (C), internal memory (M), interactions realized by control between the control granule and memory granule by means of c-granules generated by control for interactions with the external environment [c-granules with parts: M, link l-2 (l-3) and hunk H-2 (H-3)] as well as with other than memory internal parts of the agent (c-granule with parts: M, link l-1, and hunk H-1).

**Fig. 9:**
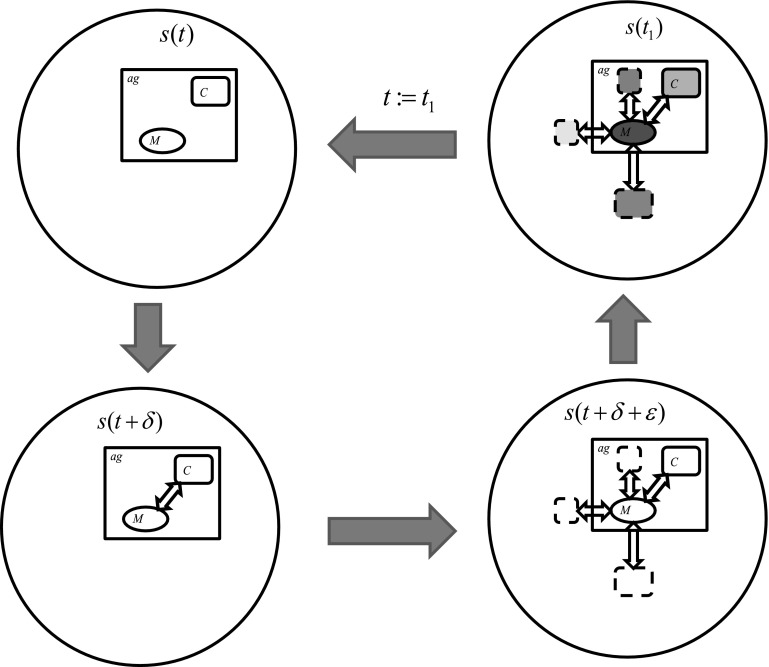
Basic control cycle of agent

In Fig. 9 the basic control cycle of agent is illustrated. In the first stage the actual interactions between control and memory are established. Next, the relevant for a given moment of (agent) time c-granules are established.The agent is planning using them for interactions with the external environment and with the agent internal parts. After this the interactions are initiated and their results are recorded in the internal memory (M) of the agent. As soon as the recording is finalized the agent control starts a new cycle.

It is worthwhile mentioning that contrary to the existing computation models realized by Turing machine the results of interactions can be only predicted by the agent control but the prediction can be in general different from the results of real interactions between agent and the environment due to uncertainty, e.g., unpredictable interactions in the environment. In particular, this is the consequence of uncertain information about the environment which the agent has due to bounds on available resources, e.g., available (or discovered so far) for agent sensors necessary for perception agent strategies.

**Fig. 10:**
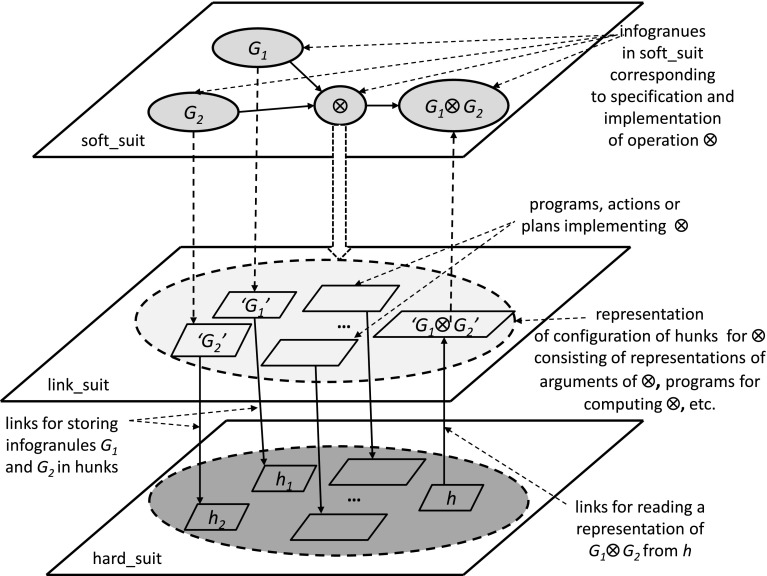
Explanation of roles of different suits of a c-granule for operation $\otimes$.

In Fig. 10 we illustrate how the abstract definition of operation from soft_suit interacts with other suits of c-granule. It is necessary to distinguish two cases. In the first case, the results of operation realized by interaction of hunks are consistent with the specification in the link_suit. In the second case, the result specified in the soft suit can be treated only as an estimation of the real one which may be different due to the unpredictable interactions in the hard_suit.

The point of view that the interactive computations on complex granules are progressing due to interactions with the physical world have are important for Natural Computing too. The agent-observer trying to understand such computations is dependent on the physical world. This argument is supported by the following point of view (see Deutsch et al. 2000, p. 268):

It seems that we have no choice but to recognize the dependence of our mathematical knowledge (...) on physics, and that being so, it is time to abandon the classical view of computation as a purely logical notion independent of that of computation as a physical process.

**Fig. 11:**
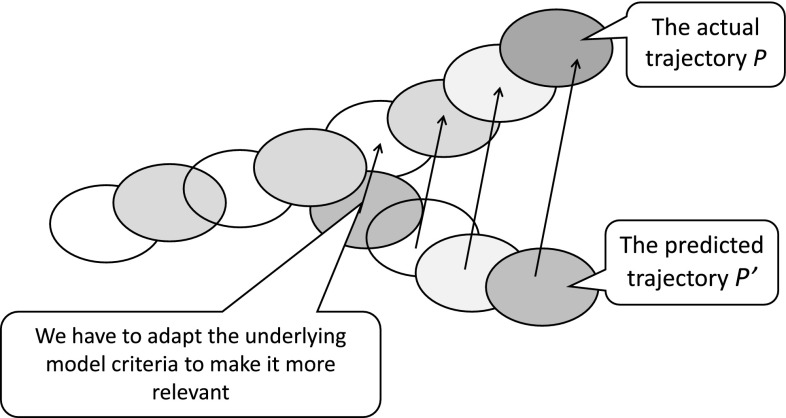
Adaptation of trajectory approximations

The agent hypotheses about the models of computations can be verified only through interactions within the physical world. These models should be adaptively changed when deviations of the predicted from the perceived real trajectories of computations are becoming significant (see Fig. 11).

The issues discussed in this section are raising a question about the control of interactive granular computations. In the next section we emphasize importance of the risk management by the agent control.

## Risk Management in IIS

Since the very beginning, all human activities were done at risk of failure. Recent years have shown the low quality of risk management in areas such as finance, economics, and many others. In this context, improvement in the risk management has a particular importance for the further development of complex systems. The importance of risk management illustrates the following example from financial sector. Many of financial risk management experts consider Basel II rules^2^ as a causal factor in the credit bubble prior to the 2007-2008 collapse. Namely, in Basel II one of the principal factors of financial risk management was

outsourced to companies that were not subject to supervision, credit rating agencies.

Of course, now we do have a new “improved” version of Basel II, called Basel III. However, according to an OECD^3^ the medium-term impact of Basel III implementation on GDP growth is negative and estimated in the range of $-0.05\%$ *to* $-0.15\%$ *per year* (see also Slovik and Cournède 2011).

On the basis of experience in many areas, we have now many valuable studies on different approaches to risk management. Currently, the dominant terminology is determined by the standards of ISO 31K [1]. However, the logic of inferences in risk management is dominated by the statistical paradigms, especially by Bayesian data analysis initiated about 300 years ago by Bayes, and regression data analysis initiated by about 200 years ago by Legendre and Gauss. On this basis, resulted many detailed methodologies specific for different fields. A classic example is the risk management methodology in the banking sector, based on the recommendations of Basel II standards for risk management mathematical models (Shevchenko 2011). The current dominant statistical approach is not satisfactory because it does not give effective tools for inferences about the vague concepts and relations between them (see the included before sentences by L. Valiant).

A particularly important example of the risk management vague concept relation is the relation of a cause - effect relationships between various events. It should be noted that the concept of risk in ISO 31K is defined as *the effect of uncertainty on objectives*. Thus, by definition, the vagueness is also an essential part of the risk concept. To paraphrase the motto of this study by Judea Pearl, we can say that traditional statistical approach to risk management inference *is strong in devising ways of describing data and inferring distributional parameters from sample*. However, in practice risk management inference requires two additional ingredients (see the motto of this article):

- a science-friendly language for articulating risk management knowledge, and
- a mathematical machinery for processing that knowledge, combining it with data and drawing new risk management conclusions about a phenomenon.

Adding both mentioned above components is an extremely difficult task and binds to the core of AI research very accurately specified by the Turing test. With regard to our applications, properly adapted version of the test boils down to the fact that on the basis of a “conversation” with a hidden risk management expert and a hidden machine one will not be able to distinguish who is the man and who is the machine.

We propose to extend the statistical paradigm by adding the two discussed components for designing of the high quality risk management systems supported by IIS.

For the risk management in IIS one of the most important task is to develop strategies for inducing approximations of vague complex concepts making it possible to check their satisfiability (to a degree). A typical example of such vague concept is the statement of the form: “now we do have very risky situation”. The starting point for development of strategies for inducing approximations of such vague complex is based on observation that the activation of actions performed by agents is based on satisfiability of such concepts.

**Fig. 12:**
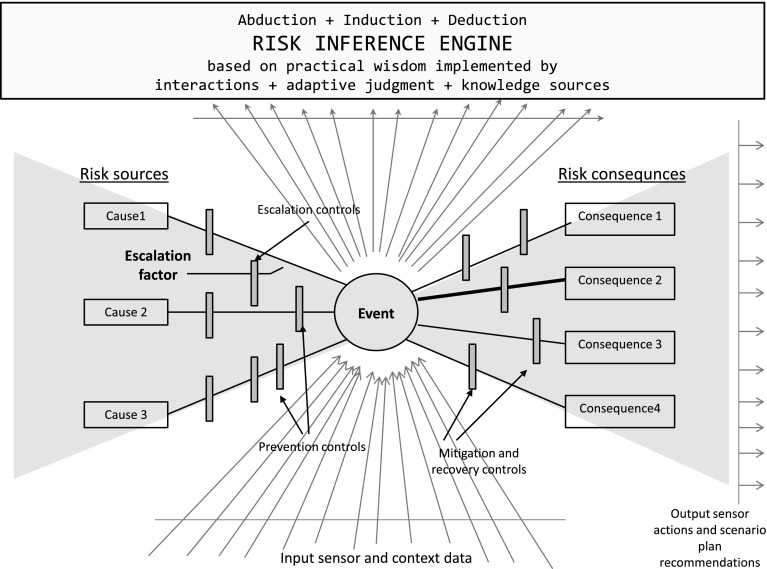
Bow-tie diagram

These vague complex concepts are represented by the agent hierarchy of needs. In risk management one should consider a variety of complex vague concepts and relations between them as well as reasoning schemes related, e.g., to the bow-tie diagram (see Fig. 12).

Let us explain the bow-tie diagram using the chess game. Of course the chess game is a very simple example. In practice the game could be much more complex. The bow-tie diagram has 3 basic parts:

1. concepts from risk sources,
2. current situation description represented by a hierarchy of concepts defined by the input sensors and context data,
3. concepts from risk consequences.

To make the next move in chess game the player should understood the current situation. To do this, he or she should use the domain knowledge representation (especially, related to the domain of risk management) and apply the relevant inference rules to the current situation description (see parts 1 and 2) enriched by knowledge about the history of moves. Based on the knowledge about possible sources of risk (expressed in part 1) and features of moves history, one should identify the prioritized list of hypotheses about the opposite player strategy. If the opposite player strategy is identified then it is much easier to win. This kind of inference leading to a list of the most likely to be true hypotheses for the opposite player strategy, is called abduction. This *is a form of logical inference that goes from observation to a hypothesis that accounts for the reliable data (observation) and seeks to explain relevant evidence* (by Wikipedia http://en.wikipedia.org/wiki/Abductive_reasoning). In the following step, the best possible next move should be proposed on the basis of the list of hypotheses for the opposite player strategy. For the chess game, one can generate the tree of all possible $n$-moves and propose the best next move using some well known algorithms (such as $minimax$, $alpha-beta$, $A-star$ (Pearl 1984). In real life applications, such trees theoretically could be generated using the part of risk ontology related to consequences (part 3). If these trees are becoming huge then using relevant abduction inference one can try to identify constraints helping to make searching for the best next move in such trees feasible.

**Fig. 13:**
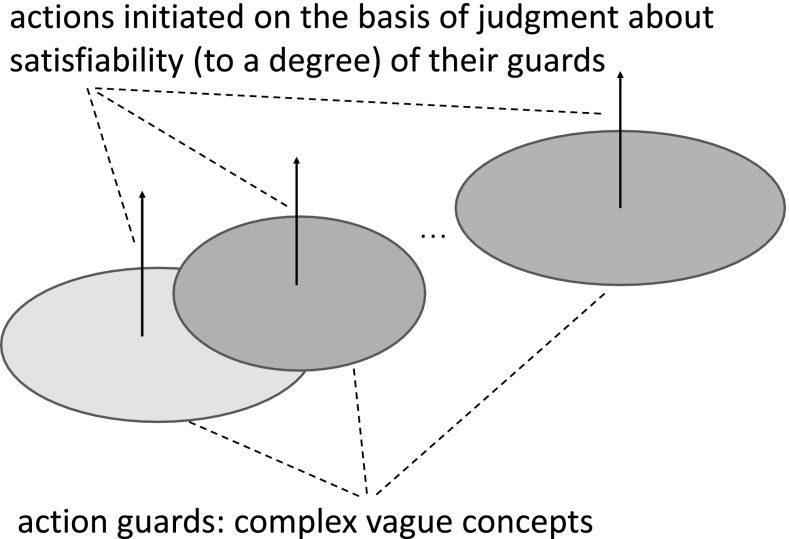
Games based on complex vague concepts

One can consider the mentioned above tasks of approximation of vague complex concepts initiating actions as the complex game discovery task (see Fig. 13) from data and domain knowledge. The agents use the discovered games for achieving their targets in the environment. The discovery process often is based on hierarchical learning supported by domain knowledge Jankowski (2015), Bazan (2008). It is also worthwhile mentioning that such games are evolving in time (drifting in time) together with data and knowledge about the approximated concepts and the relevant strategies for adaptation of games used by agents are required. These adaptive strategies are used to control the behavior of agents toward achieving by them targets. Note that also these strategies should be learned from available uncertain data and domain knowledge.

The discussed concepts such as interactive computation and adaptive judgment are among the basic ingredient elements in the Wisdom Technology (WisTech) [10], Jankowski (2015). Let us mention here the WisTech meta-equation:

2 $$\begin{aligned} \text{ WISDOM }&= \text{ INTERACTIONS }\nonumber \\&+ \text{ADAPTIVE } \text{JUDGMENT }\nonumber \\&+ \text{ KNOWLEDGE }. \end{aligned}$$

An extension of the rough set approach on interactive computations realized by IIS is one of the current challenges.

## Conclusions

The approach for modeling interactive computations based on c-granules was presented and its importance for the risk managements was outlined.

The presented approach seems also to be of some importance for developing computing models in different areas such as natural computing (e.g., computing models for meta-heuristics or computations models for complex processes in molecular biology), computing in distributed environments under uncertainty realized by multi-agent systems (e.g., in social computing), modeling of computations for feature extraction (constructive induction) for approximation of complex vague concepts, hierarchical learning, discovery of planning strategies or strategies for coalition formation by IIS as well as for approximate reasoning about interactive computations based on such computing models.

In our research, we plan to further develop the foundations of interactive computations based on c-granules toward tools for modeling and analysis of computations in Natural Computing (Rozenberg et al. 2012), Wisdom Web of Things (Zhong et al. 2013) or Cyber-Physical Systems (Lamnabhi-Lagarrigue et al. 2014).

## Footnotes

1 see, e.g., http://en.wikipedia.org/wiki/Vagueness, http://people.seas.harvard.edu/~valiant/researchinterests.htm.

2 see http://en.wikipedia.org/wiki/Basel_Committee_on_Banking_Supervision.

3 see http://en.wikipedia.org/wiki/Basel_III.
